# Effectiveness of the International Phytosanitary Standard ISPM No. 15 on Reducing Wood Borer Infestation Rates in Wood Packaging Material Entering the United States

**DOI:** 10.1371/journal.pone.0096611

**Published:** 2014-05-14

**Authors:** Robert A. Haack, Kerry O. Britton, Eckehard G. Brockerhoff, Joseph F. Cavey, Lynn J. Garrett, Mark Kimberley, Frank Lowenstein, Amelia Nuding, Lars J. Olson, James Turner, Kathryn N. Vasilaky

**Affiliations:** 1 United States Department of Agriculture, Forest Service, Northern Research Station, Lansing, Michigan, United States of America; 2 United States Department of Agriculture, Forest Service, Research and Development, Arlington, Virginia, United States of America; 3 Scion (NZ Forest Research Institute), Christchurch, New Zealand; 4 Better Border Biosecurity, New Zealand; 5 United States Department of Agriculture, Animal and Plant Health Inspection Service, Plant Protection and Quarantine, Plant Health Programs, National Identification Services, Riverdale, Maryland, United States of America; 6 United States Department of Agriculture, Animal and Plant Health Inspection Service, Plant Protection and Quarantine, Center for Plant Health Science and Technology, Raleigh, North Carolina, United States of America; 7 Scion (New Zealand Forest Research Institute), Rotorua, New Zealand; 8 New England Forestry Foundation, Littleton, Massachusetts, United States of America; 9 National Center for Ecological Analysis and Synthesis, University of California Santa Barbara, Santa Barbara, California, United States of America; 10 University of Maryland, Agricultural and Resource Economics, College Park, Maryland, United States of America; 11 AgResearch Ltd., Ruakura Research Centre, Hamilton, New Zealand; 12 Earth Institute and International Research Institute for Climate and Society, Columbia University, New York, New York, United States of America; University of Kent, United Kingdom

## Abstract

Numerous bark- and wood-infesting insects have been introduced to new countries by international trade where some have caused severe environmental and economic damage. Wood packaging material (WPM), such as pallets, is one of the high risk pathways for the introduction of wood pests. International recognition of this risk resulted in adoption of International Standards for Phytosanitary Measures No. 15 (ISPM15) in 2002, which provides treatment standards for WPM used in international trade. ISPM15 was originally developed by members of the International Plant Protection Convention to “practically eliminate” the risk of international transport of most bark and wood pests via WPM. The United States (US) implemented ISPM15 in three phases during 2005–2006. We compared pest interception rates of WPM inspected at US ports before and after US implementation of ISPM15 using the US Department of Agriculture AQIM (Agriculture Quarantine Inspection Monitoring) database. Analyses of records from 2003–2009 indicated that WPM infestation rates declined 36–52% following ISPM15 implementation, with results varying in statistical significance depending on the selected starting parameters. Power analyses of the AQIM data indicated there was at least a 95% chance of detecting a statistically significant reduction in infestation rates if they dropped by 90% post-ISPM15, but the probability fell as the impact of ISPM15 lessened. We discuss several factors that could have reduced the apparent impact of ISPM15 on lowering WPM infestation levels, and suggest ways that ISPM15 could be improved. The paucity of international interception data impeded our ability to conduct more thorough analyses of the impact of ISPM15, and demonstrates the need for well-planned sampling programs before and after implementation of major phytosanitary policies so that their effectiveness can be assessed. We also present summary data for bark- and wood-boring insects intercepted on WPM at US ports during 1984–2008.

## Introduction

International trade has been responsible for the inadvertent introduction of many exotic (nonnative) insect pests and plant pathogens, of which several have become highly invasive and caused serious environmental and economic impacts to multiple habitats worldwide [1]–[7]. In recent years, introductions of several particularly damaging wood-infesting insects and pathogens in the United States (US) have focused public and regulatory attention on the pathways that transport these pests [8]–[12].

Wood-feeding insects are commonly associated with wood packaging material (WPM), which includes items such as pallets, crates, and dunnage (wood used to brace cargo). Packaging for overseas shipments is commonly constructed from wood because it is relatively inexpensive, generally abundant, renewable, and easily manufactured and repaired. Unfortunately, wood used to construct WPM can be infested with a wide variety of bark and wood pests and thereby serve as a pathway for pest movement. Wood-feeding insects can also be transported in logs, lumber, fuelwood, live plants, and various manufactured wood articles [12]–[16].

As international trade volumes soared in recent decades, many countries became concerned about repeated introductions of invasive forest insects and disease organisms, such as Asian longhorned beetle, *Anoplophora glabripennis* (Motschulsky) (Coleoptera: Cerambycidae), and pinewood nematode, *Bursaphelenchus xylophilus* (Steiner et Buhrer) Nickle (Nematoda: Aphelenchoididae), as well as the WPM pathway that often vectors these pests. In response, members of the International Plant Protection Convention (IPPC) developed and adopted International Standards for Phytosanitary Measures No. 15 (ISPM15) in 2002, which provided details on approved phytosanitary treatments for WPM used in international trade [17]. A core value of these international standards is the harmonization of national regulations, which facilitates trade. The original stated goal of ISPM15 in 2002 was to “practically eliminate the risk for most quarantine pests and significantly reduce the risk from a number of other pests” by means of either heat treatment or methyl bromide fumigation of WPM [17]. ISPM15 was slightly revised in 2006 [18], and in 2009 the IPPC adopted several important changes such as lengthening the fumigation exposure time, requiring WPM to be made from debarked wood, requiring debarking prior to fumigation, and specifying tolerance limits on the maximum allowable size for individual patches of residual bark [19]. In addition, the goal of ISPM15 was reworded in 2009 to read as follows “to reduce significantly the risk of introduction and spread of most quarantine pests” associated with WPM [19]. The next version of ISPM15 was published in 2011 but consisted simply of changes in text formatting [20]. The newest version of ISPM15 was approved in 2013 and formally adopted heat treatment using dielectric heating (e.g. microwave) along with the corresponding treatment code DH [21]. More than 78 countries (considering the European Union as 27 countries) have implemented ISPM 15 through October 2013. It is important to recognize that the ISPM15 standards can be applied to wood from any tree species, including tropical and boreal species, as well as softwoods (conifers) and hardwoods (angiosperms).

The United States implemented ISPM15 in three phases over a 10-month period from 16 September 2005 to 5 July 2006. On 16 September 2005 the United States implemented Phase 1, which consisted of officially informing importers and the appropriate National Plant Protection Organization of the exporting country if live pests were found in WPM or if the WPM was not marked in compliance with ISPM15. Phase 2 began on 1 February 2006 and required that all WPM entering the United States (except from Canada) meet ISPM15 treatment standards and be marked accordingly. As part of Phase 2, noncompliant shipments and WPM could be denied entry to the United States, or if feasible, the noncompliant WPM would be removed from the shipment and exported at the expense of the importer, and thereby allow the imported products to enter the United States. Phase 3 began on 5 July 2006 and continues to the present and requires that noncompliant WPM and the associated commodities be immediately exported, usually returning it to the country of origin [22].

The objective of the present paper was to compare pre- and post-ISPM15 infestation rates of WPM associated with imports entering the United States. In this paper, we use the term “infestation rate” to refer to the percentage of consignments with WPM in which live pests were found in WPM when the imported consignments were inspected on arrival at US ports. We expected that if the data from the pre- and post-ISPM15 surveys were comparable then we could estimate the effect that ISPM15 had on WPM infestation rates. Further, we anticipated that implementation of ISPM15 would substantially reduce the number and frequency of live pests in WPM because the supporting documents that accompanied the early drafts of ISPM15 indicated that the proposed treatments for WPM were highly effective against many wood-associated insects and fungal pathogens [23]–[24]. The use of interception data for this purpose seemed acceptable because interception records are among the few datasets available that provide insights into the identity and relative infestation rate of pests associated with traded commodities and WPM [8], [15], [25]. We were able to find one large US dataset with interception data that had been collected in a standardized manner both pre- and post-ISPM15, which upon analysis indicated a moderate decline in pest interceptions on WPM after ISPM15 implementation.

Documenting the actual level of effectiveness of an international policy such as ISPM15 and evaluating the suitability of existing data for such an analysis is important for at least three reasons. First, it is important for determining the level of phytosanitary risk still associated with WPM and whether further revisions to ISPM15 are needed, or if individual countries may wish to require additional measures based on a pest risk assessment. Second, it is essential for estimating and understanding the economic costs and benefits of the implemented policy. And, third, it provides insights into the types of data that should be collected in advance of future international standards. For example, the recent approval of ISPM 36 in 2012 [26], which deals with plants for planting, provided such an opportunity.

### ISPM15 Standards

To fulfill the requirements of ISPM15, WPM used in international trade must be marked (stamped) in a specific way to indicate that the WPM was subjected to an approved phytosanitary treatment [21]. The official mark includes the IPPC logo, a 2-letter country code indicating in which country the wood was treated, a producer code to indicate the treatment provider, and a treatment code to specify the treatment used, such as HT for heat treatment or MB for methyl bromide fumigation [21]. Each version of ISPM15 has provided more details on how the wood treatments should be conducted, and even more details were added to the 2013 version [21]. After research showed that bark- and wood-infesting insects, both primary and secondary colonizers, could infest and develop in wood after treatment, especially when bark was present [27]–[28], a debarking requirement for WPM was added in 2009. The tolerance limits for residual bark specified that pieces of bark could remain on WPM after debarking if individually they were either less than 3 cm in width (regardless of their length) or if they were greater than 3 cm wide but less than 50 square centimeters in total surface area [19]–[21]. The debarking requirement was not yet in place during the period of time analyzed in the present study.

### Pests Commonly Associated with WPM

The principal bark- and wood-boring insects of quarantine concern for the United States include insects in the beetle (Coleoptera) families Buprestidae, Cerambycidae, Curculionidae (including Platypodinae and Scolytinae); the woodwasp family Siricidae (Hymenoptera), and the moth (Lepidoptera) families Cossidae and Sesiidae. Elsewhere in the world there are many other wood pests of concern to specific countries, including species of powderpost beetles (Bostrichidae, including Lyctinae), wood-boring flies (Diptera), termites (Isoptera), as well as wood-decay fungi and nematodes [29]–[32]. It is important to note that many powderpost beetles and termites are secondary colonizers of treated wood, and therefore are rarely the target pests when ISPM15 treatments are applied to newly constructed WPM.

### International Pest Interception Databases

Several countries maintain databases of plant pests that are intercepted at their ports of entry, including maritime ports, airports, and international border crossings. For example, long-term pest interception databases have been maintained by governments and plant protection organizations in Australia, Canada, Chile, Europe and North Africa (by the European and Mediterranean Plant Protection Organization, EPPO), Mexico, New Zealand, and the United States. Typically, inspectors target high-risk products or pathways, rather than conduct random surveys. In addition, interception records are usually included in a country’s database only when pests are found although there are exceptions (such as the AQIM database used in the present study).

### Earlier Surveys for WPM-Associated Pests

A comprehensive review of the literature, involving online literature searches as well as direct contacts with several plant protection organizations worldwide, provided a limited number of estimates of WPM infestation rates from before implementation of ISPM15 [33]–[35] and after [28], [36] (Table 1). In general, the pre-ISPM15 surveys were expressed on a consignment basis, such as all WPM in a single shipping container. In contrast, the sampling units used in the two post-ISPM15 surveys were individual WPM items such as a single pallet or a single piece of dunnage. Therefore, the results of these pre- and post-ISPM15 surveys were not directly comparable. Nevertheless, in the pre-ISPM15 surveys, WPM infestation rates ranged from a high of 4.3% of containerized maritime consignments [33] to a low of 0.06% for air cargo consignments [35]. By contrast, in the two post-ISPM15 surveys that involved mostly maritime containerized cargo, infestation rates of individual WPM items ranged from 0.1% [28] to 0.5% [36] (Table 1).

**Table 1:** Summary data for the incidence of live insects found in association with WPM during surveys of imported goods that were conducted before or after implementation of ISPM 15 in various countries.

Country wheresurvey wasconducted	Year ofsurvey	Was survey conductedpre-ISPM15 orpost-ISPM15?	Type of portwhere survey wasconducted^a^	Sampling unitfor WPMinspected^b^	No. of wood itemsor consignmentsinspected	Percent of wood items or consignments infested with live insects^c^	Percent of inspectedconsignments or WPMitems with bark^d^	Reference
New Zealand	1989–91	Pre-ISPM15	Seaport (Con)	Consignment	1,366	4.32%	5.0%	Bulman (1992) [33]
New Zealand	1993	Pre-ISPM15	Seaport	Consignment	7,916	0.67	3.5	Bulman (1998) [34]
Australia	1997–99	Pre-ISPM15	Seaport (Con)	Consignment	56,193	0.38	5.9	Salvage (1999) [35]
Australia	1997–99	Pre-ISPM15	Seaport (Non-Con)	Consignment	1,104	0.27	5.5	Salvage (1999) [35]
Australia	1997–99	Pre-ISPM15	Airport	Consignment	62,701	0.062	1.7	Salvage (1999) [35]
Chile	2004–05	Pre-ISPM15	Seaport	Consignment	7,733	0.181	n.a.	Sánchez-Salinas (2007) [37]
Chile	2005–06	Post-ISPM15	Seaport	Consignment	3,137	0.096	n.a.	Sánchez-Salinas (2007) [37]
Australia	2005	Post-ISPM15	Seaport	Individual WPM items	19,522	0.51	8.1	Zahid et al. (2008) [36]
USA	2006	Post-ISPM15	Sea & land ports	Individual WPM items	5,945	0.11	9.4	Haack & Petrice (2009) [28]

a Seaport = maritime seaports; Con = containerized cargo; Non-Con = non-containerized cargo, also called break bulk cargo; Land ports = land border crossing between Mexico and USA.

b WPM = wood packaging material. Consignment = typically these are imported goods that are similar in nature, have a similar origin, have been packaged and shipped in a similar manner, and have arrived at a port-of-entry at the same time. WPM items = individual units such as one entire pallet, or all crating associated with one imported product, or one piece of dunnage.

c WPM infestation levels were based on values reported by the authors or on calculations based on data they included in their published papers. The infestation rate in Bulman [33] was based on the 1366 consignments that definitely contained WPM (i.e., cases, crates, pallets, and skids) as presented in Table 3 of that paper. These inspections were based on a random sample of less-than-full container loads. The rate in Bulman [34] was based on the 7916 consignments listed in Table 4 of that paper that definitely contained WPM, using the same categories listed for Bulman [33]. These inspections targeted primarily containers with full-container loads of cargo considered high-risk by the inspector and consisted of “door inspections” in which the doors at the end of the container are opened and all commodities are inspected as well as possible without removal. The values given in Salvage [35] were the published infestation levels and the containerized cargo was for less-than-full container loads; no information was given on how the consignments were selected. Values in Sánchez-Salinas [37] were for all consignments with WPM that arrived at the maritime port of San Antonio, Chile, during the 30-month inspection period (18 mo pre- and 12-mo post-ISPM15), and were based on data in Tables 8 and 10 in the thesis [37] after deleting the bostrichid data so that the analysis would be similar to our analysis of the AQIM data. Using the sample size data presented in Sánchez-Salinas [37], the calculated 47% reduction in infestation rate of WPM entering Chile after implementation of ISPM15 was not statistically significant (*P* = 0.23, Fisher’s exact test, one-tailed). Zahid et al. [36] presented actual infestation levels of individual WPM items with the ISPM15 mark that were sampled at three maritime seaports; however, the authors did not state how the WPM items were selected. Haack and Petrice [28] presented infestation levels of individual WPM items sampled at five seaports and one land border crossing during a 2-wk period at each port; the sampled WPM items were from containers that were selected for inspection during normal port operations.

d Percentage values for the frequency at which bark was found is based on the same sampling protocol described above in footnote c.

We found only one publication, a master’s thesis [37], which compared interception data that had been collected in a similar manner both before and after implementation of ISPM15. In this study, the author summarized the insect interceptions on WPM that were associated with 10,870 consignments that arrived at the maritime port of San Antonio, Chile during the 18 months immediately before (7733 consignments) and 12 months immediately after (3137) implementation of ISPM15 in Chile. The interception data were expressed on a consignment basis, and included live bark- and wood-infesting insects that were intercepted in WPM. Overall, data from Sánchez-Salinas [37] indicated that the infestation rate of WPM entering Chile fell 47% after ISPM15 was implemented (Table 1).

### USDA Pest Interception Databases

The US Department of Agriculture (USDA) Animal and Plant Health Inspection Service (APHIS) maintains two major databases for records of pest interceptions on imported goods at US ports: AQIM (Agriculture Quarantine Inspection Monitoring) and PestID (Pest Interception Database, which was formerly called Port Information Network or PIN). AQIM is a statistically based inspection program based on random sampling of imported shipments at selected US ports. AQIM was designed to monitor the approach rate of agricultural risks on different pathways, and consists of daily or weekly random sampling of international cargo, mail, vehicles, and passenger baggage [38]. WPM was first targeted for inspection in AQIM in 2003 and usually consisted of sampling two containers per week at each of more than 40 participating US ports. Sample selection occurs randomly among commodities known to have associated WPM using a statistically robust stratified sampling plan. Infestation data for WPM are recorded on a consignment basis based on the number of distinct consignments within each of the sampled shipping containers. For each pest interception in AQIM, information is recorded on all pests found to the lowest taxonomic level possible (usually family, genus or species), as well as on the type of cargo inspected, type of WPM present, compliance with ISPM15 marking, and the presence or absence of bark on the WPM. For WPM, all plant pests found are recorded in AQIM, including both bark- and wood-infesting insects as well as those that inadvertently contaminated or “hitchhiked” with the shipment. Negative data, where no pests are found, are also recorded by consignment in AQIM, which allows the calculation of infestation rates (contrary to other interception data where negative inspections are typically not documented).

PestID includes interceptions records of all classes of plant pests intercepted at over 300 ports of entry in the United States, including bark- and wood-infesting insects found in association with WPM. As of January 2014, there were more than 2.5 million interception records in PestID that were recorded since 1984 when what is now known as PestID started as a computerized database. PestID records include information on the identity of intercepted pests, the commodity involved and its country of origin, date and place of interception, and many other details associated with the shipment and inspection such as whether the intercepted pest was associated with WPM. Unlike AQIM, however, PestID does not include information on shipments where no pests were found, and the inspections are not random, but are targeted at specific products, pathways, or countries.

There are other challenges when attempting to interpret PestID data. For example, although APHIS issues inspection guidelines for certain commodities [39], much work prioritization is left to the discretion of experienced, local personnel at the individual ports. As a result, for some commodities and items like WPM, the percentage of arriving shipments inspected can vary over time and among ports. Inspectors may target shipments based on a perceived risk of infestation for certain commodities from particular countries of origin and shippers. Additionally, priority inspection targets vary among ports due to the profile of work to be performed. For example, port inspectors who must clear large volumes of perishable fruits, vegetables, or cut flowers will likely spend less effort inspecting WPM associated with machine parts or quarry products than inspectors at ports that do not receive many perishables. Other limitations on the utility of PestID include: 1) that the data cannot provide an estimate of the number of pests arriving because not all shipments are inspected and inspectors may stop looking at a particular consignment once the first quarantine pest is found, 2) data on many intercepted pests that were classified as “non-quarantine significant pest ” taxa (e.g., cosmopolitan species or species that were regarded to be of low risk) were not included in PestID until March 2009, and 3) variation over time in the numbers of inspectors and their focus likely affected the numbers and kinds of pests that were intercepted.

Although the sampling protocols used in PestID are not random, PestID data are still useful in identifying the most common types of pests arriving in the United States, their countries of origin, and the commodities and pathways they were most often associated with [13], [16], [40]. We report various PestID summary statistics below.

## Methods

### AQIM Data Analyses

We analyzed AQIM records where WPM was recorded for a 6-year period from October 2003 through September 2009. This period was chosen because it begins when APHIS started inspecting WPM as part of the AQIM program and ended in 2009, which was the year when several changes were made to ISPM15 [19]. Therefore, the data analyzed during the post-ISPM15 period in the present paper were collected during a period with consistent regulations. We excluded Canadian shipments from our analysis because the United States did not require Canadian WPM to meet ISPM15 standards during the sampling period. The policy of limited inspection on shipments from Canada is largely because most bark- and wood-infesting insects native to Canada are also native to the United States and because the long shared and largely forested border between the two countries presents no barriers to the migration of native or non-native insects. For example, about 97% of bark and ambrosia beetle species (Scolytinae) native to Canada are also native to the United States [41]. Similarly, we excluded all Chinese imports from our AQIM analysis because as of 17 December 1998, which was nearly six years prior to US implementation of ISPM15, the United States began regulating WPM from China in response to the rapidly increasing frequency of pest interceptions on Chinese WPM in the 1990s and the discoveries of Asian longhorned beetle infestations in New York in 1996 and Illinois in 1998 [8], [42]–[43]. This 1998 regulation on WPM [43] only affected exports from China to the United States. During the period from 1999 until US implementation of ISPM15, noncompliant Chinese shipments were typically fumigated at US ports, whereas after US implementation of ISPM15 most noncompliant shipments were sent back China. In addition, given that Mexico was the origin of more AQIM records than any other country (34% of all AQIM records during the 6-year study period, and 41% of the dataset after removal of the Canadian and Chinese records), we analyzed the remaining data both with and without the records from Mexico. The large number of Mexican consignments in the AQIM database was because several US-Mexico border crossings participated in AQIM program.

In our analyses, we tested separately the initial dates of Phase 1 (16 September 2005) and Phase 3 (5 July 2006) as the division points between pre- and post-implementation of ISPM15. For each date, we tested two scenarios: 1) exclusion of all data related to Canadian and Chinese imports, and 2) exclusion of all data related to Canadian, Chinese, as well as Mexican imports (for reasons explained above). We constructed a 2×2 contingency table for each scenario, comparing pre- and post-ISPM15 infestation rates of WPM, and analyzed each for statistical significance using Fisher’s exact test (right-sided probability, PROC FREQ) [44]. We used a significance level of α = 0.1 because infestation rates of WPM are typically low and we wished to reduce the likelihood of committing a Type II error (i.e., a false negative). We also calculated the power of our analysis to detect large reductions in pest infestation rates using presumed treatment effectiveness levels for ISPM15 of 50%, 70% and 90% mortality of the WPM-associated quarantine pests (PROC POWER) [44]. These results, tested with α = 0.05 and 0.1, would indicate the probability of detecting a 50%, 70% and 90% change in infestation rate had one occurred. When calculating the post-ISPM15 infestation rates in the above analyses, we only used data for those consignments in which the WPM was apparently compliant with ISPM15, i.e., stamped with the ISPM15 mark. We also calculated on an annual basis the percent of inspected consignments in which the WPM had the proper ISPM15 mark after US implementation of ISPM15 (2005–2009), and analyzed the data with nonlinear regression (PROC NLIN) [44]. In addition, we used methods similar to those described above to compare the pre- and post-ISPM15 infestation rates of WPM from the single country of Italy, which was the country of origin for the most borer interceptions on WPM that entered the United States during 1985–2000 [8].

### PestID Data Analyses

PestID data cannot be used to statistically analyze for the effects of ISPM15 on interception rates because the data are collected in a nonrandom manner and the number of inspections where no pests are found is not recorded. Nevertheless, we did extract all interceptions of bark- and wood-boring insects in PestID from the 25-year period 1984 through 2008 to demonstrate changes over time in the types of borers being intercepted, the countries of origin, and the imported commodities most often associated with wood pests. As noted earlier, we recognize that the PestID data can be influenced by many factors such as changes in interception policies, staffing, etc. We restricted the dataset to those families of wood borers that were consistently targeted during port inspections over the 25-year period: Buprestidae, Cerambycidae, Cossidae, Curculionidae (including Platypodinae and Scolytinae), Sesiidae, and Siricidae. For records where the imported commodity was reported, we assigned the commodity to one of several trade sectors according to the Global Trade Analysis Project (GTAP) [45]–[46]. For example, some of the common GTAP sectors that we used included fabricated metal products (e.g., ironware, metalware, tubes, and wire), primary metals (e.g., aluminum, iron, and steel), machinery and equipment, quarry products (e.g., granite, marble, and slate), and fruit and vegetables.

## Results

### AQIM

Overall, there were 34,981 inspection records of consignments that contained WPM in the AQIM database from October 2003 through September 2009. These consignments came from 137 countries, with the top 15 countries being Mexico (33.7%), Italy (14.2%), Canada (13.4%), Netherlands (4.4%), China (4.1%), Costa Rica (3.8%), Guatemala (2.9%), Ecuador (1.9%), Dominican Republic (1.7%), Brazil (1.7%), India (1.6%), Spain (1.6%), Turkey (1.5%), Honduras (1.3%), and Germany (0.9%). WPM-associated insects of quarantine significance were intercepted on only 50 of the 34,981 consignments (0.14%). These 50 interceptions were associated with imports from 16 countries that represented 4 world regions, including 16 records from 4 Asian countries, 17 from 7 European countries, 14 from 2 Central American countries (including Mexico), and 3 records from 3 South American countries (Table S1). No wood pests were found on the Canadian imports. The 50 insect interceptions consisted of 26 interceptions of Cerambycidae, 22 Scolytinae, 1 Platypodinae, and 1 Cossidae (Table S1).

**Figure 1:**
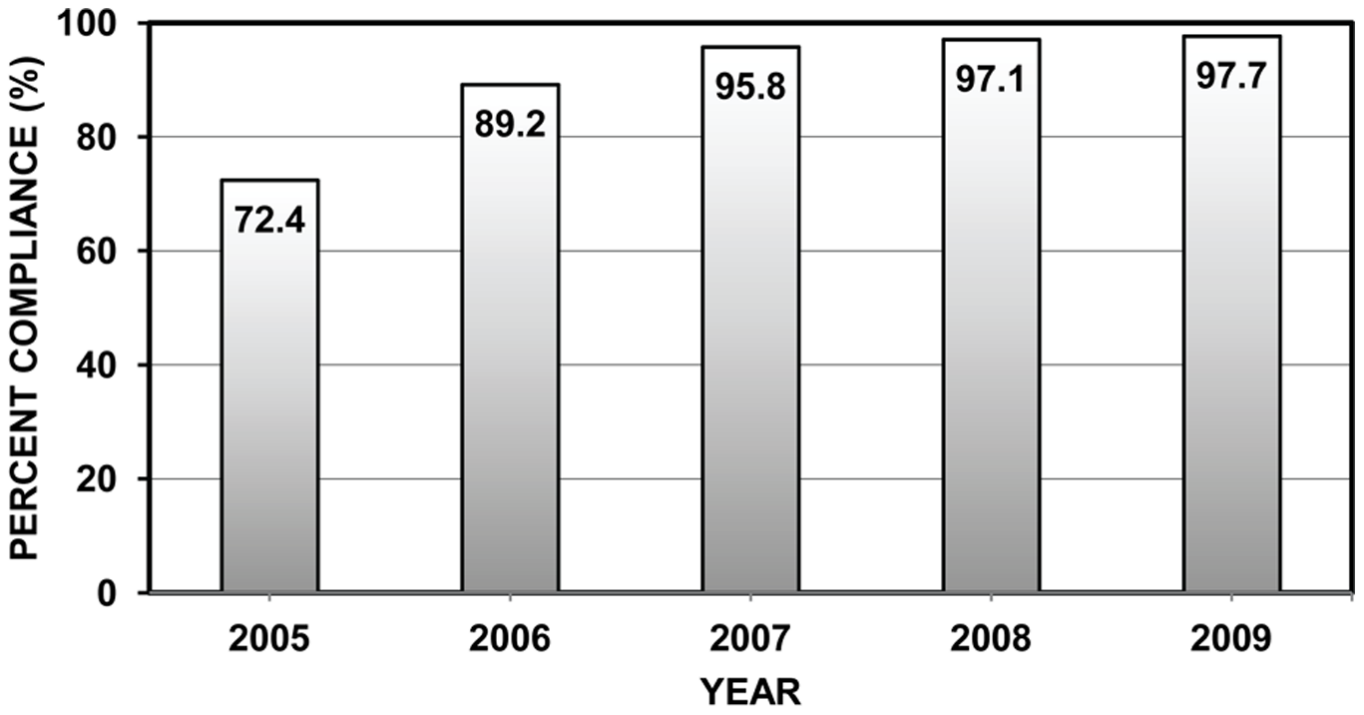
Percent of imported consignments entering the US with ISPM15-compliant WPM by year. Percent of consignments with wood packaging material (WPM) entering the United States that was compliant with ISPM15 (i.e., the WPM was stamped with the official ISPM15 mark) after the United States implemented ISPM15 in September 2005. Data are presented on an annual basis for the period 2005–2009; however, the percentage value for 2005 was based on data from October-December 2005, while the 2009 value was based on data from January-September 2009. Percentage values were based on the AQIM database for all countries except Canada (N = 23,551 consignments). Using nonlinear regression (PROC NLIN [44]) the following model was fit to the above data: Percent Compliance = 100–27.42 × exp(−0.0884 × years_since_2005), R^2^ = 0.992, F_1,3_ = 361, P = 0.00032.

The percentage of consignments with compliant WPM (i.e., WPM with the official ISPM15 mark) entering the United States increased steadily from 2005 to 2009 (Figure 1). Overall, for all countries (after excluding data from Canada), WPM associated with 21,993 of 23,551 consignments was marked correctly from September 2005 to October 2009 (93%). When the data were presented on an annual basis, the percentage of compliant WPM entering the United States increased significantly (P<0.001) from about 72% in 2005 to nearly 98% in 2009 (Figure 1). Similarly, for the 4084 post-ISPM15 Canadian consignments with WPM, which were not required to meet ISPM15 standards when shipping to the United States, the percentage of WPM with the ISPM15 mark nevertheless also increased over time but was consistently and expectedly much lower than the rest of the world (Figure 1), being about 6% in 2005, 12% in 2007, and 24% in 2009.

The actual pre- and post-ISPM15 infestation rates of WPM entering the United States ranged from 0.17 to 0.25% pre-ISPM15 to 0.11 to 0.12% post-ISPM15 (Table 2). Recall that the post-ISPM15 values were calculated using only consignments with compliant (marked) WPM. Based on the above pre- and post-ISPM15 infestation rate values, the infestation rate of WPM entering the United States declined by 36–52% after implementation of ISPM15, depending on which countries and division dates were used in the analyses (Table 2). Of the four scenarios tested, only one resulted in a reduction that was statistically significant at the *P* = 0.1 level, while the other three scenarios had *P* values that ranged from 0.111 to 0.127 (Table 2). In general, there was a greater reduction in infestation rates when the Phase 1 date was used to divide the pre- and post-ISPM15 sampling periods, or when data from Mexico (in addition to Canada and China) were deleted prior to analysis (Table 2).

**Table 2:** Percent reduction in infestation rate on a consignment basis for bark- and wood-infesting insects in WPM associated with US imports after implementation of ISPM15, using two different dates to separate pre- and post-ISPM15 and different country groupings.

	No. consignments^b^					
		Infested/Not infested		Infestation rate^d^		
Countries excluded^a^	Total	Pre-ISPM15	Post-ISPM15	Pre	Post	Percent reduction (*P* = )^e^
Phase 1 division (before versus after 15 September 2005)^c^						
CA, CN	27185	12/6315	24/20834	0.1897%	0.1151%	39.3% (0.111)
CA, CN, MX	16475	9/3664	15/12787	0.2456	0.1173	52.2% (0.067)
Phase 3 division (before versus after 4 July 2006)						
CA, CN	27185	17/9917	19/17232	0.1711	0.1101	35.7% (0.124)
CA, CN, MX	16475	12/6028	12/10423	0.1987	0.1150	42.1% (0.127)

a All countries were included in the analyses except various combinations of Canada (CA), China (CN), and Mexico (MX). See text for details.

b Total = number of consignments analyzed after dropping the data from the selected countries listed. Pre- and Post-ISPM15 values, in order of appearance, are the number of pre-ISPM15 consignments with and without pests, and the number of post-ISPM15 consignments with and without pests. These were the values used in the contingency tables.

c Analyses were conducted on AQIM records with WPM from 1 October 2003 through 30 September 2009. The United States implemented the first phase of ISPM15 on 16 September 2005 (Phase 1) and the final phase on 5 July 2006 (Phase 3).

d Infestation rates were based on the table values presented here under ‘No. consignments.’ For example: (12/6327) *100 = 0.1897%.

e Percent reduction is based on the difference between the pre- and post-ISPM15 infestation rates as given in this table. The formula used was [(Pre – Post) *100/Pre]. The *P* values were based on 2 x 2 contingency tables using the values presented in this table and analyzed with the Fisher’s exact test (right-sided).

Results of the power analyses for data associated with the Phase-1 dividing date (Table 2), indicated that with the available sample size there was nearly a 96% probability of detecting a statistically significant reduction in infestation rates of WPM if ISPM15 had reduced the number of infested shipments by 90%, depending on the scenario tested (with and without data from Mexico) and the alpha level selected (0.1 or 0.05) (Table 3). Similarly, there was a 70–90% probability of detecting a significant reduction in infestation rate if ISPM15 reduced the occurrence of live pests by 70%, but only a 38–62% probability if ISPM15 reduced infestation levels by just 50% (Table 3).

**Table 3:** Probability of detecting a statistically significant reduction in infestation rates if actual rates of infestation were reduced by the designated percentages after ISPM15 implementation based on post-hoc power analyses that used the observed pre-ISPM15 infestation rate and actual pre- and post-ISPM15 sample sizes from the Phase-1 scenarios presented in Table 2.

				Post-hoc Power to detect a significant reduction in infestation rates of consignments with WPM	
Presumed percentreduction in numberof consignments withinfested WPM	Observed pre-ISPM15 infestation rate basedon data in Table 2 (%)	Approximate post- ISPM15infestation rate based on datafrom columns 1 and 2 inthis Table [1−(Col 1×Col 2)]	Pre-/post-ISPM15sample sizes analyzedbased on data fromTable 2	α = 0.1	α = 0.05
Data from Canada and China excluded					
90%	0.1897%	0.01897	6327/20858	99.6%	98.8%
70	0.1897	0.05691	6327/20858	89.8	81.7
50	0.1897	0.09485	6327/20858	62.4	48.1
Data from Canada, China, and Mexico excluded					
90%	0.2456%	0.02456	3673/12802	98.0%	95.5%
70	0. 2456	0.07368	3673/12802	81.3	70.1
50	0. 2456	0.1229	3673/12802	52.4	38.1

In the case of Italy (N = 5256 WPM records), the WPM infestation rate declined by 80% when the Phase-1 date was used to divide the pre- and post-ISPM15 periods (0.36% to 0.07% infestation rate; *P* = 0.04). Similarly the infestation rate fell by 55% when using the Phase 3 date to separate pre- and post-ISPM15 periods (0.20% to 0.09% infestation rate; *P* = 0.24).

### PestID

**Figure 2:**
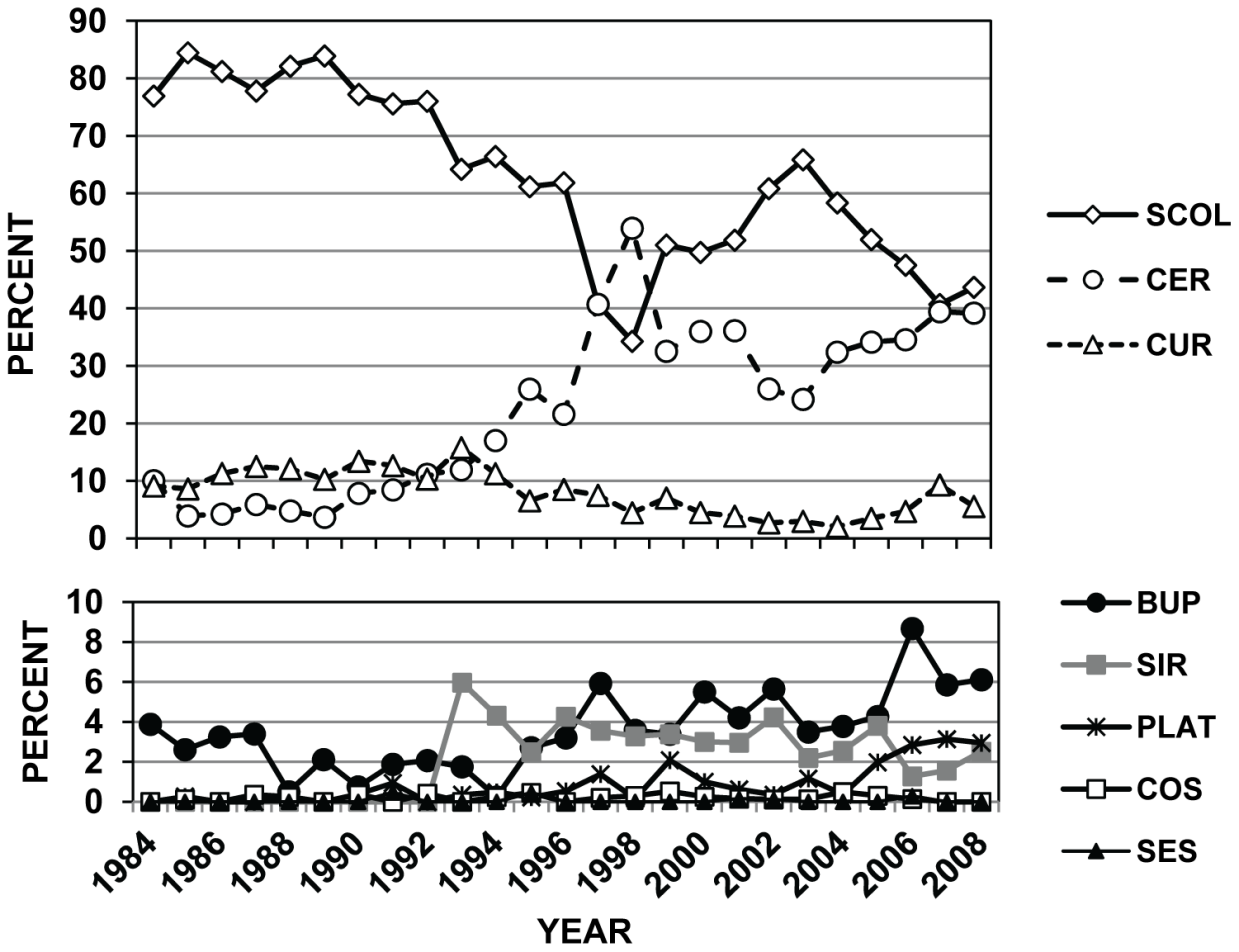
Changes over time in major groups of wood-infesting insects entering the US in WPM. Annual changes in the percent composition of eight major groups of WPM-infesting insects intercepted at US ports during 1984–2008. Percent values were calculated for each year based on the total number of WPM-interceptions in PestID for those eight groups of insects in each particular year (N = 13,768 interceptions for all 25 years). Abbreviations are: BUP = Buprestidae, CER = Cerambycidae, COS = Cossidae, CUR = Curculionidae (not including Platypodinae and Scolytinae), PLAT = Platypodinae, SCOL = Scolytinae, SES = Sesiidae, and SIR = Siricidae.

There were 13,768 PestID interception records for bark- and wood-boring insects on WPM at US ports during the 25-year period of 1984–2008. Of these 13,768 records, 36 were on shipments from Canada, 1551 from China, and 3284 from Mexico (Table 4). Of the major families and subfamilies of insects represented by these 13,768 interceptions, Scolytinae were the most commonly intercepted wood pest when considering imports from all countries (8286/13,768 = 60.2%; Table 4). Cerambycidae (longhorned beetles) were the next most commonly intercepted wood pest (25.3%). When the data were viewed annually, Scolytinae were the most commonly intercepted wood pest for nearly the entire 25-yr period, representing a low of 34% of the intercepted borers in 1998 to a high of over 84% in 1985 (Figure 2). The number of cerambycid interception records has increased dramatically since the mid-1990s (Figure 2), coinciding with increased emphasis by regulatory agencies on the WPM pathway after the discovery of Asian longhorned beetle in New York in 1996 [8], [10].

**Table 4:** Summary data for the 13,768 interceptions of bark- and wood-infesting insects in WPM at US ports during 1984–2008 by country of origin and insect family or subfamily (Source = USDA APHIS PestID database).

Order	Selected countries					Next top 10 countries in decreasing order after Mexico and China									
Family	All	CA^b^	CN	MX	Other^c^	IT	DE	ES	TR	BE	FR	IN	RU	UK	PT
**Coleoptera**															
Buprestidae	553	3	46	98	397	40	9	81	154	18	4	15	2	2	4
Cerambycidae	3483	13	952	379	2041	670	75	135	275	51	71	59	85	11	22
Curculionidae^a^	981	0	48	183	696	181	83	55	22	56	36	4	18	71	12
Platypodinae	141	0	26	34	79	1	0	0	0	1	2	8	0	1	1
Scolytinae	8286	19	428	2581	4995	1322	715	533	180	316	255	225	137	137	178
**Hymenoptera**															
Siricidae	292	1	44	8	236	103	45	19	9	4	19	1	0	0	1
**Lepidoptera**															
Cossidae	25	0	6	1	16	1	0	1	1	2	2	1	1	0	0
Sesiidae	7	0	1	0	5	1	0	0	1	0	0	0	0	0	0
**Total**	13,768	36	1551	3284	8465	2319	927	824	642	448	388	314	243	222	218

a The values for the weevil family Curculionidae do not include the two subfamilies Platypodinae and Scolytinae, which are listed separately.

b Country codes: BE Belgium, CA Canada, CN China, DE Germany, ES Spain, FR France, GR Greece, IN India, IT Italy, MX Mexico, PT Portugal, RU Russia, and TR Turkey.

c The category “Other” did not include 432 records for which no country of origin was listed.

**Figure 3:**
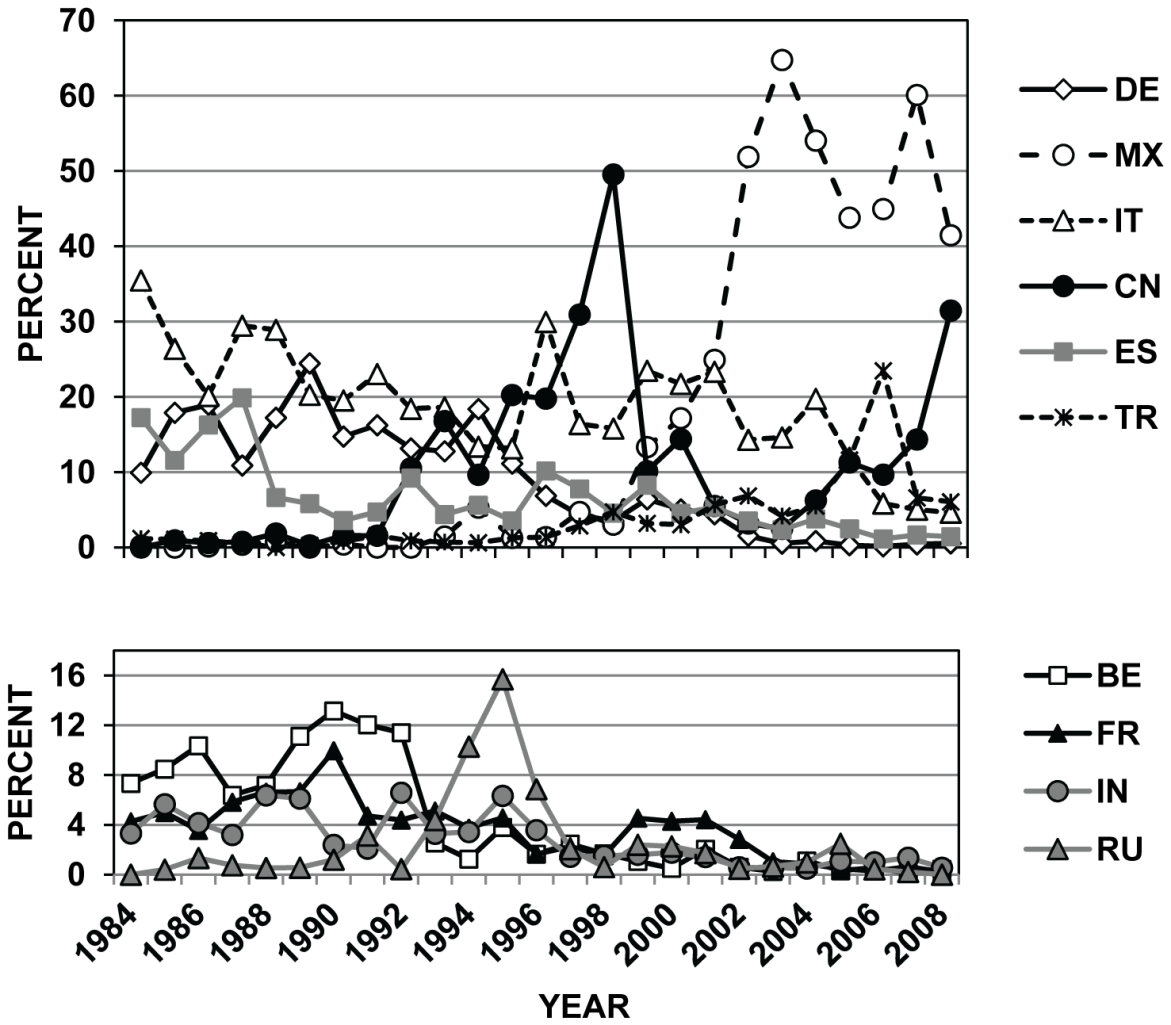
Changes over time for wood-infesting insects entering the US in WPM by country of origin. Annual changes in the percent composition of the top 10 countries of origin for bark and wood-infesting insects intercepted in WPM that were associated with imports to the United States during 1984–2008. Values were calculated for each year based on the total number of WPM interceptions in PestID where the country of origin was recorded (N = 13,328 interceptions. No country of origin was listed for 440 records). Abbreviations are: BE = Belgium, CN = China (not including 24 interceptions from Hong Kong and 42 from Taiwan for all years), DE = Germany, ES = Spain, FR = France, IN = India, IT = Italy, MX = Mexico, RU = Russia (including 32 interceptions coded as Soviet Union from 1984 to 1993), and TR = Turkey.

When considering the 12 countries that were the origin of most of the intercepted wood pests over the 25-yr period, Cerambycidae were the most frequently intercepted family from 3 of the 12 countries (China, Italy, and Turkey) and Scolytinae from the other 9 countries (Belgium, France, Germany, India, Mexico, Portugal, Russia, Spain; and United Kingdom; Table 4). The largest numbers of wood-associated pest interceptions at US ports during 1984–2008 were from Mexico (23.9% = 3284/13,768), Italy (16.8%), and China (11.3%; Table 4). The relative ranking of countries that were the source for infested WPM has changed dramatically in recent decades (Figure 3). For example, European countries (e.g., Belgium, Germany, Italy, and Spain) were the source for most US interceptions on WPM in the 1980s, while China, Mexico, and Turkey were the main sources of wood pests in the 2000s (Figure 3).

**Figure 4:**
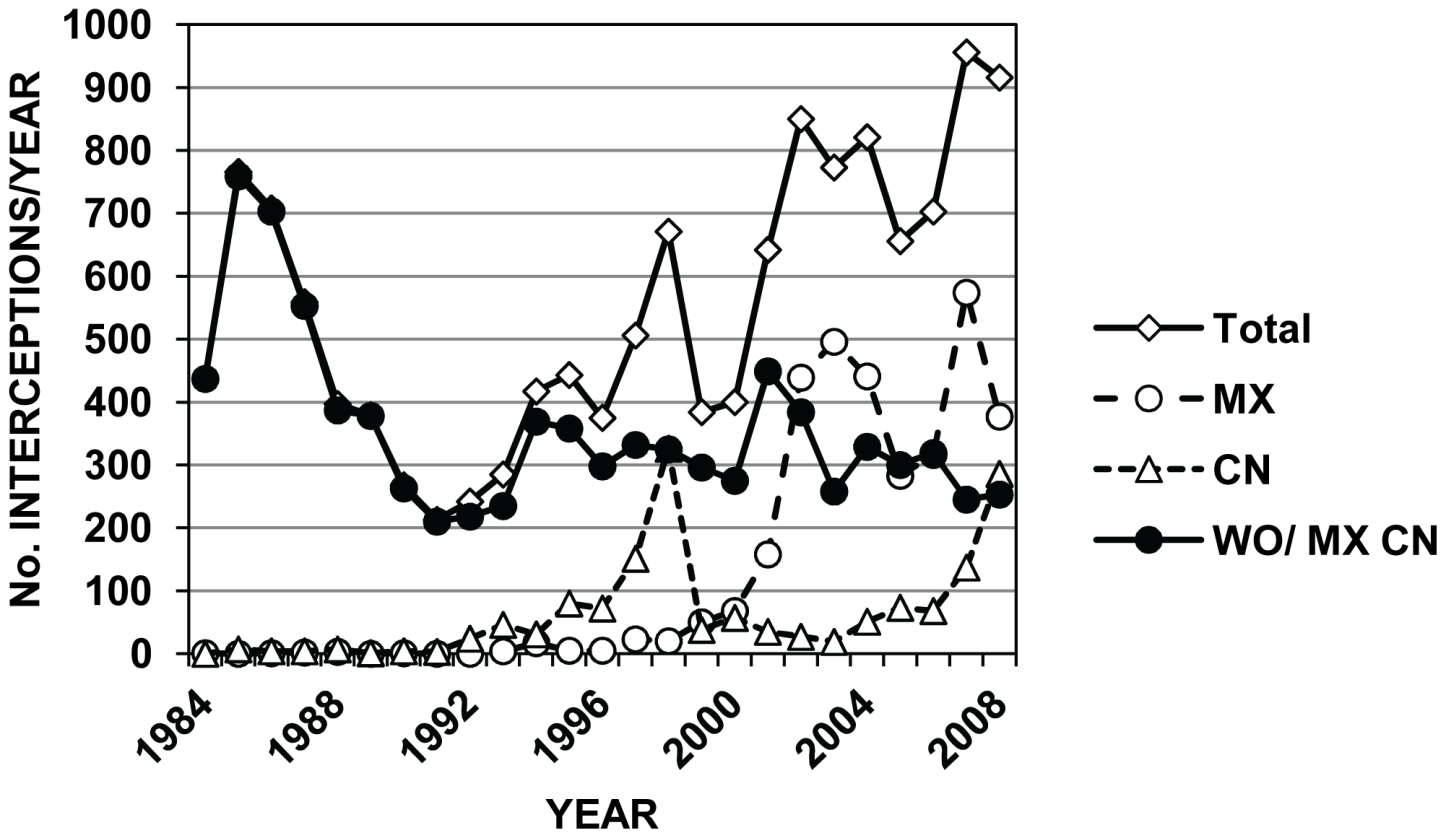
Number of wood-infesting insect interceptions made in WPM at US ports by year. Annual number of WPM-associated pest interceptions at US ports during 1984–2008 in the PestID database by year for all countries combined (Total) as well as individually for China (CN) and Mexico (MX), and the total minus the number for Mexico and China (WO/MX CN).

**Figure 5:**
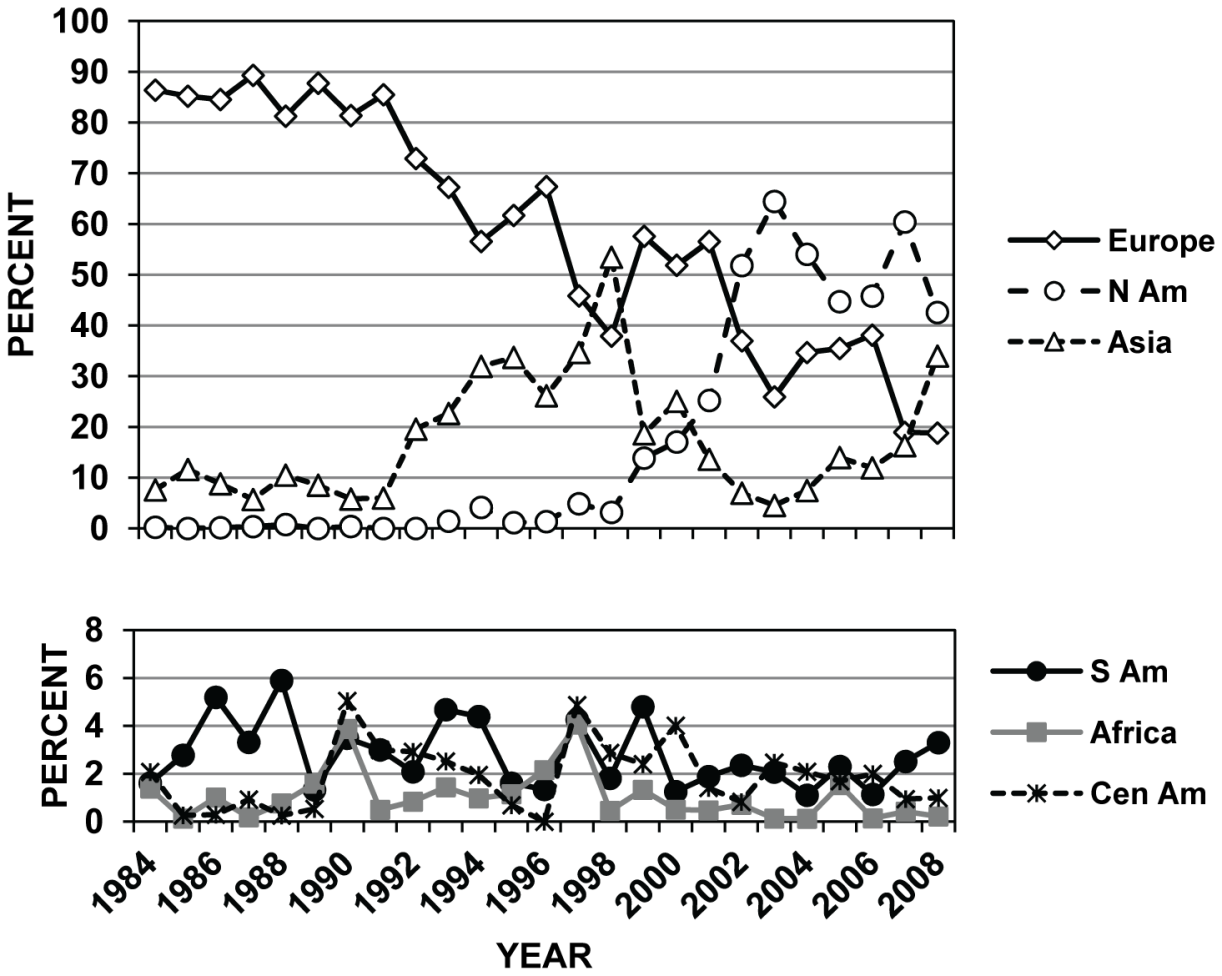
Changes over time for wood-infesting insects entering the US in WPM by world region. Annual changes in the percent composition of six major world regions as the origin for bark and wood-infesting insects intercepted in WPM associated with imports to the United States during 1984–2008. Values were calculated for each year based on the total number of WPM-interceptions in PestID where the world region of origin was recorded (N = 13,607 interceptions. No world region was listed for 161 records). Abbreviations are: Cen Am = Central America+Caribbean Islands, Europe = Europe, including Russia and Turkey, N Am = Canada+Mexico, and S Am = South America. No data are shown for the relatively few interceptions made on imports from Australia, New Zealand, Philippines, and countries in the Middle East.

These changes in country rankings probably reflect more shifts in US trading partners, new national inspection policies, and initiation of new international trade agreements, rather than dramatic changes in infestation status of WPM from individual countries. For example, Mexico was the origin of very few recorded interceptions on WPM in the 1980s and early 1990s, but after initiation of NAFTA (North American Free Trade Agreement) in 1994 and greater focus by APHIS inspectors on wood pests from Mexico in the late 1990s, imports and pest interceptions from Mexico increased markedly [8], [47] (Figure 3). Similarly, interceptions from China were very low (0–7 per year) from 1984 until 1991, grew rapidly through 1998, and then declined sharply in 1999 after the United States required only China to treat its WPM prior to export to the United States [43] (Figure 3). However, in recent years, interceptions on Chinese WPM have increased again along with strong increases in imports from China [8], [48] (Figure 3). The annual numbers of WPM-associated pest interceptions at US ports during 1984–2008 are shown in Figure 4 for all countries (including Canada) as well as for China and Mexico separately. When the interception data were viewed at the scale of world regions, there was a clear shift from Europe being the primary source of WPM-pests from the mid-1980s to the mid-1990s to Asia and North America (primarily Mexico) from the mid-1990s to present (Figure 5).

**Figure 6:**
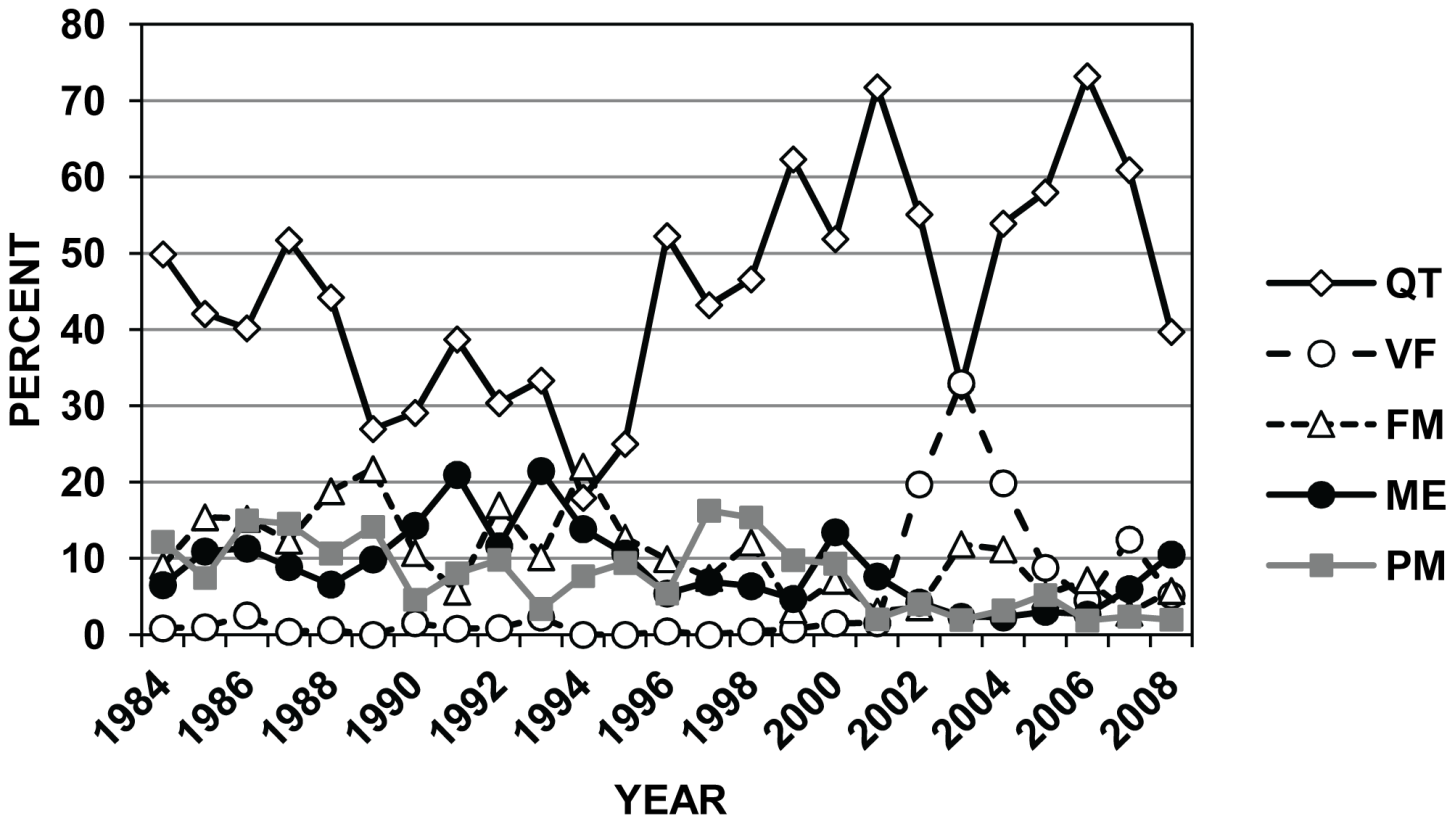
Changes over time for wood-infesting insects entering the US in WPM by associated commodity class. Annual changes in the percent composition of five major commodity classes of imports that entered the United States during 1984–2008 and were associated with interceptions of bark and wood-infesting insects in WPM. Values were calculated for each year based on the total number of WPM-interceptions in PestID where the imported commodity was recorded (N = 8661 interceptions. No imported commodity was listed for 5107 records). Abbreviations are: FM = fabricated metal products (e.g., ironware, metalware, tubes, and wire), ME = machinery and equipment, PM = primary metals (e.g., aluminum, iron, and steel), QT = quarry products and tiles (e.g., granite, marble, slate, and tiles), and VF = vegetables and fruit.

The commodity associated with the infested WPM was listed on 8661 of the 13,768 interception records (63%). The two commodities that were most often associated with borer-infested WPM were tiles (2291 of 8661 records, 26.5%) and quarry products (1765 records, 20.4%). Changes in the relative rankings of the five major GTAP commodity sectors that were associated with borer-infested WPM are shown in Figure 6. Overall, the sector that includes quarry products and tiles was the sector associated with the most WPM-associated pests in 24 of the 25-year sampling period, representing 18–73% of the interceptions in any single year (Figure 6). Similarly, the range in annual percentage contributions over the 25-year period for WPM-associated pests were 3–22% for fabricated metal products, 2–21% for machinery and equipment, 2–16% for primary metals, and 0–33% for vegetables and fruit (Figure 6).

## Discussion

To make valid comparisons of pest interception rates before and after implementation of a major policy like ISPM15 it would be best to have a large multi-year dataset that was collected under uniform conditions. The AQIM dataset largely meets these goals in that we were able to assemble a 6-year dataset (about 2-years before and 4 years after ISPM15 implementation) that contained nearly 35,000 records, which were collected using uniform inspection procedures in a statistically random manner.

Overall, we estimated that infestation rates of WPM entering the United States declined by 36–52% to about 0.11% after ISPM15 implementation (Table 2). In general, these values are very similar to the 47% reduction reported for WPM entering Chile where infestation rates declined from 0.181% pre-ISPM15 to 0.096% post-ISPM15 (Table 1). The earliest of the surveys listed in Table 1 was conducted on maritime containerized cargo in New Zealand during 1989–1991 [33]. In that study, conducted more than 12–14 years before New Zealand implemented ISPM15, the infestation rate of WPM was about 4.3% (Table 1). If we consider 4.3% to be representative of the WPM infestation rates worldwide during the early 1990s, then our current estimate of about 0.11% reflects more than a 97% reduction in infestation rates. Nevertheless, given that the original stated goal of ISPM15 was to “practically eliminate the risk for most quarantine pests” in WPM [17], a more significant reduction than 36–52% was anticipated in the interception rate of WPM-associated pests after ISPM15 implementation in the United States. Similarly, our power analysis indicated that the AQIM sample size was sufficiently large to have nearly a 96% probability of detecting a 90% reduction in WPM infestation rates.

As noted in the introduction, WPM used in international trade is now stamped with the official ISPM15 mark after treatment. However, as reported in this study and by others [28], [36]–[37], at times live wood pests are still found in ISPM15-marked WPM. There are several reasons that could help explain why live insects are occasionally found in ISPM15-marked WPM and why there was not a greater reduction in infestation rate of WPM after implementation of ISPM15.

### Possible Factors Influencing the Impact of ISPM15

#### Pest tolerance of the treatment

Finding live bark- and wood-infesting insects in treated WPM could indicate that some wood pests can survive the ISPM15 treatments. This could have occurred because the pinewood nematode was used as the target pest in the development of the ISPM15 heat-treatment schedule, which requires a minimum of 56°C for a minimum of 30 min (56/30) as measured at the core of the wood [32], [49]. Nevertheless, several scientific papers suggesting that 56/30 would kill many, but not all, species of wood-inhabiting insects and pathogens were listed in the original draft ISPM15 that was distributed for country consultation in 2001 [23]. Given this supporting documentation, the 56/30 heat schedule was “chosen in consideration of the wide range of pests for which this combination is documented to be lethal and a commercially feasible treatment” [23]. In addition, it should be noted that the 2001 draft ISPM15 recognized that “some pests are known to have a higher thermal tolerance” and thus could survive 56/30 [23]. Considering the thousands of wood-infesting insect species worldwide [32], it is possible that some of the live insects encountered in heat-treated WPM did survive treatment.

With respect to heat treatment, for example, Mushrow et al. [50] indicated that 56/30 was adequate to kill larvae and pupae of brown spruce longhorned beetle, *Tetropium fuscum* (Fabricius) (Coleoptera: Cerambycidae). However, in the case of emerald ash borer, *Agrilus planipennis* Fairmaire (Coleoptera: Buprestidae), a small percentage of larvae survived treatments that bracketed 56/30, but none of these studies matched the 56/30 requirements exactly [51]–[54] (Table 5). For example, in the studies listed in Table 5, the authors generally did not record the wood temperature at the wood core and usually did not test 56/30 specifically. Similar variation in response to heat treatment has been documented in various wood-colonizing fungi [55]–[56]. Moreover, Sobek et al. [57] noted that slow heating rates in laboratory experiments can activate heat shock proteins in emerald ash borer larvae, making them more tolerant of heat treatment, but the authors did state that this would seldom occur in commercial kilns. ISPM15 does not stipulate a minimum chamber air temperature or heating rate, but rather states the minimum endpoint of 56/30. Further testing of 56/30 and other potential treatments against a broader range of bark and wood-infesting insects could clarify the role of treatment tolerance in the continuing low level of WPM infestation. In addition, as a measure of establishment risk, it would be important to determine whether any insects that survive treatment can complete development and reproduce. Nevertheless, given that during heat treatment, temperatures at the surface of the WPM would exceed the core temperature where ISPM15 measurements are made [52], borers residing close to the surface would therefore experience temperatures that surpass the required minimum temperature of ISPM15 and thus should suffer higher mortality.

**Table 5:** Summary details for four studies where the effects of heat treatment were tested on emerald ash borer (EAB), *Agrilus planipennis*, and how these studies compared with ISPM15 standards.

	Publication			
Parameter	McCullough et al. 2007 [51]	Myers et al. 2009 [53]	Nzokou et al. 2008 [52]	Goebel et al. 2010 [54]
Type of heat chamber	Drying oven	Drying oven and heat chamber	Laboratory kiln	Small outdoor kiln
Type of wood tested	Bark and wood chips	Split firewood with bark	Logs with bark	Whole and split firewood with bark
Location of temperature probe	Chamber air	Standard depth of 3.5 cm belowbark	Log center and at 1 cm depth	Standard depth of 2.5 cm
Number of temperature probes used	1 probe measuring chamber air temperature	1 probe in each piece of wood	2 probes per log	3 probes per load of 100 pieces
Temperatures tested	40, 45, 50, 55, 60°C	50, 55, 60, 65°C	50, 55, 60, 65°C	46, 56°C
Times tested	20 and 120 min	30 and 60 min	30 min	30 and 60 min
Sample size per treatment	12 larvae	24–32 wood pieces	4 logs	100 wood pieces per load, 1 load per temperature
Starting and set point chamber temperature	Preheated to test temperature	Preheated to 80°C then loweredto 5°C above targettemperature	Preheated to 82°C and maintained at that temperature	65°C set point; started at 2°C
Major differences from ISPM15 heat treatment standard	Tested wood chips, monitored air temperature, did not test 56°C for 30 min	Did not monitor woodtemperature at center, did nottest 56°C for 30 min	Did not test 56°C for30 min	Did not monitor wood temperature at center
Major findings	Some EAB survival in all treatments except 60/120	Low or no EAB survival at 60and 65°C for both 30 and60 min	Low EAB survival at 55/30 and 60/30; alldied at 65/30	Some EAB survived all treatments, but few at 56/30 and 56/60

Similarly, there are several factors that affect the efficacy of methyl bromide fumigation in wood. One factor is the depth to which the fumigant can penetrate wood, especially green (not dried) wood. For example, in a study using green pine (*Pinus*) roundwood with bark and with the cut ends sealed, Cross [58] reported that lethal concentrations of methyl bromide did not reach much beyond 10 cm into the wood. This finding was addressed in the 2009 revision of ISPM15 [19], which specified that methyl bromide treatment should not be used on WPM that exceeds 20 cm in cross section. Therefore, it is possible that some live insects encountered in fumigated WPM could represent situations where lethal levels of the fumigant did not reach the insect. In the WPM survey conducted at six US ports in 2006, live borers were found in both heat-treated and fumigated WPM [28]. In general, fumigants should be able to pass easily through larval galleries to reach most wood borer larvae, but the permeability of their galleries can be influenced by the presence of frass (insect boring dust and feces), which is packed tightly in the galleries of some borers [59]. The 2009 requirement to debark WPM prior to fumigation [19] was intended to improve fumigant penetration, which was an improvement not reflected in the data analyzed in the present paper. In addition, it is important to note that methyl bromide is no longer used within the European Union to fumigate WPM.

#### Unintentional noncompliance

It is possible that managers at treatment facilities attempt to treat WPM according to ISPM15 but for some reason the minimum required dose of fumigant or heat is not appropriately or not evenly applied in the treatment chamber. There are many factors that can bring about such unintentional noncompliance. For example, a manager may follow the treatment schedules precisely based on sensors within the chamber, but because of cold pockets or uneven distribution of the fumigant not all wood is treated equally. For heat treatment, ISPM15 specifies that temperature probes need to be carefully inserted to the core of the largest wood pieces present in the chamber during each treatment cycle. If the probes do not reach the center of the wood or if a probe is not well sealed from the ambient air then the target temperature of 56°C will be indicated sooner than it should. To obtain accurate readings all equipment must be calibrated and working properly. In addition, fans are often needed in chambers to help circulate the fumigant or heated air, and the individual WPM items must be properly stacked to ensure good air flow. Each of these factors, as well as many others (e.g., presence of bark, cross-sectional size of wood pieces), could result in reduced mortality of wood pests during treatment. For these reasons, many countries require treatment facilities be certified by an approved accreditation agency, such as the American Lumber Standards Committee (www.alsc.org) in the United States. Presumably these types of deficiencies would be more likely in countries that do not require third party accreditation. Several new procedural recommendations were listed in Annex 1 of the 2013 version of ISPM 15 [21] to address the above factors when heat treating or fumigating WPM, and these changes should further improve the efficacy of ISPM15.

#### Fraud

Unfortunately, intentional noncompliance or fraud does occur. This happens when the ISPM15 mark is knowingly applied to WPM that has not been treated or not properly treated. Widespread usage of WPM with fraudulent marks, especially if infested, would reduce the apparent impact that ISPM15 has had on reducing WPM infestation rates. Companies found guilty of mark fraud in the United States can be fined and suspended from the WPM certification program.

#### Post-treatment colonization of WPM

Some bark- and wood-boring insects can colonize and complete development in WPM after treatment, especially when bark is present. For example, Evans [27] and Haack and Petrice [28] found that several species of borers (Cerambycidae and Scolytinae) colonized and completed development in heat-treated logs and boards that retained varying amounts of bark. Moreover, Haack and Petrice [28] noted that the size and shape of individual bark patches greatly influenced borer colonization and subsequent larval survival. Given the above results, it is possible that some of the live insects found in treated WPM could have resulted from post-treatment colonization of treated WPM. It is important to note, however, that the above two studies [27], [28] were conducted in a manner to facilitate post-treatment infestation of WPM, given that the treated WPM was placed back into forested habitats soon after treatment where bark and wood-infesting insect populations were expected to be high. Nevertheless, these findings, among others, were used to justify the 2009 revision of ISPM15 that stipulated the maximum size of any individual patch of residual bark [19].

#### Data issues in AQIM

Two important features of the AQIM program are that pest interceptions on WPM are recorded 1) on a consignment basis rather than for individual WPM items, and 2) as simply presence/absence rather than the number of individual pest organisms found. Because there can be wide variation in the number of WPM items in a single consignment, as well as the percentage of WPM items infested per consignment and the number of pests present in a single piece of infested WPM, it is possible that ISPM15 actually reduced infestation rates and the numbers of live wood pests more than was revealed through our analysis of the AQIM data.

#### Policy anticipatory effect

In our analysis of the AQIM data, we found a greater reduction in the percentage of consignments entering the United States with WPM-associated pests after implementation of ISPM15 when using the Phase-1 date as the division point between pre- and post-ISPM15, as compared with using the Phase-3 date (Table 2). This finding suggests that many foreign shippers of products to the United States started using ISPM15-compliant WPM soon after initiation of Phase 1 rather than waiting until Phase 3 when full implementation began. This could easily have occurred because there was worldwide discussion about ISPM15 for several years before it was initially implemented in the United States in 2005. For example, the draft version of ISPM15 was circulated worldwide for country consultation in 2001 [23], and later adopted in 2002 [17]. In addition, the global community had advance warning that the United States had plans to implement ISPM15 given that the United States published its intentions in a proposed rule on 20 May 2003, and later announced its final rule on 14 September 2004 [60]. Moreover, the 2004 final rule did not state that an incremental 10-month-long phase-in of ISPM15 would take place, but rather stated that the United States would fully implement ISPM15 one year later on 16 September 2005 [60]. In addition, many countries implemented ISPM15 before the United States such as New Zealand in 2003, Australia in 2004, and the European Union in 2005. Given that it is often more convenient for shippers to maintain an inventory of one type of pallet, such as ISPM15-compliant pallets, it is likely that some of the WPM entering the United States during 2003–2005 was already ISPM15-compliant. For example, as mentioned above, ISPM15-compliant WPM was associated with 24% of the Canadian consignments that entered the United States in 2009 even though Canada was not required to use ISPM15-compliant WPM when shipping to the United States, and still is not required to do so as of April 2014.

## Conclusions

Our analysis of the AQIM data indicated only a modest reduction in pest infestation rates of WPM entering the United States following implementation of ISPM15, declining from about 0.2% (for the 2 years pre-ISPM) to about 0.1% (for the 4 years following ISPM15). AQIM is well designed, but given the low infestation rate of WPM, even during the years immediately before ISPM15 was implemented in the United States, this dataset lacks the power to detect modest reductions in infestation rates with confidence. We determined that the AQIM dataset had about 96% power to detect a 90% reduction in pest levels post-ISPM15, but to detect more modest levels of reduction with high confidence would have required more intensive sampling. Thus we conclude that either ISPM15, as implemented through 2009, did not have the anticipated high level of impact on infestation rates of WPM entering the United States or that the impact of ISPM15 began to influence WPM infestation rates earlier than 2003 for which we do not have adequate data. As mentioned earlier, if the 4.3% WPM infestation rate reported from surveys conducted in New Zealand during 1989–1991 [33] were typical of the 1990s, then the current infestation rate of 0.1% would represent a major reduction of about 97%. The current AQIM program could be strengthened by increasing sample size or by making adjustments in the sampling protocol, such as recording both the number of WPM items in each consignment and the number of WPM items that were infested with live pests.

Although a 0.1% infestation rate of WPM appears very low (1 in 1000 consignments), this value should be considered in terms of the total number of consignments entering the United States each year. For example, it was estimated that about 25 million shipping containers entered the United States in 2013 [61] and that about 52% of containers have WPM [24]. Based on those two figures, and assuming the cargo in each container represents a single consignment, an infestation rate of 0.1% of 13 million containers ( = 52% of 25 million) would represent 13,000 containers per year entering the United States with live wood pests. Such a large number of infested consignments could lead to new pest establishments given that the probability of establishment increases with pathway volume [15], [62].

It is also important to remember that ISPM15 is not static. Several changes have been made since the first version of ISPM15 was published in 2002, and more changes will likely follow. For example, the recent changes in 2009 that dealt with debarking, fumigation, and size limits on residual bark [19] should further reduce the occurrence of live pests in WPM. Even more improvements were made in the 2013 version of ISPM15 [21]. Future analyses of the AQIM database for the 2009–2013 period would be useful to document if further reductions in WPM infestation rates occurred as a result of the changes made to ISPM15 in 2009.

The relatively low number of published surveys of WPM-associated pests before and after implementation of ISPM15, as well as the variable methods that were used in each survey, demonstrates the need to conduct surveys before and after implementation of major phytosanitary standards that are comparable to assess policy effectiveness. Without such information it is exceedingly difficult to verify the extent to which a policy change results in the desired effects.

Nevertheless, it is commendable that the world community recognized the phytosanitary risk posed by WPM and subsequently approved ISPM15 in 2002, and has continued to improve it through regular revisions. It was likely very challenging to set treatment standards for WPM that were acceptable and achievable by most countries, given that tree species, pest species, and the availability of phytosanitary treatment facilities vary from country to country worldwide.

## Supporting Information

Table S1 **Summary data for the 50 insect interceptions made at US ports on wood packaging material (WPM) in the AQIM database during the period 2003 to 2009, including 15 interceptions made pre-ISPM15, 5 interceptions made during the US phase-in period of ISPM15, and 30 interceptions made after full implementation of ISPM15.**
(DOC)
