# Supplementary material for: Effectiveness of the International Phytosanitary Standard ISPM No. 15 on Reducing Wood Borer Infestation Rates in Wood Packaging Material Entering the United States
Source: PLoS One. 2014 May 14;9(5):e96611. doi: 10.1371/journal.pone.0096611 (PMC4020780; doi:10.1371/journal.pone.0096611)
Supplement: Table S1 — Summary data for the 50 insect interceptions made at US ports on wood packaging material (WPM) in the AQIM database during the period 2003 to 2009, including 15 interceptions made pre-ISPM15, 5 interceptions made during the US phase-in period of ISPM15, and 30 interceptions made after full implementation of ISPM15. (DOC) [file pone.0096611.s001.doc]

**Table S1. Summary data for the 50 insect interceptions made at US ports on wood packaging material (WPM) in the AQIM database during the period 2003 to 2009, including 15 interceptions made pre-ISPM15, 5 interceptions made during the US phase-in period of ISPM15, and 30 interceptions made after full implementation of ISPM15.**

| **Insects intercepted on WPM** | |  |  |  |
| --- | --- | --- | --- | --- |
| **Family ^a^** | **Lowest taxon identified** | **Continent of origin ^b^** | **WPM type** | **Imported product** |
| **Interceptions made Pre-ISPM 15 (2003-2005)** | | | | |
| Cerambycidae | *Callidium* sp. | Europe | Dunnage | Magnesium ingots |
| Cerambycidae | Cerambycidae | Asia | No data | Machine parts |
| Cerambycidae | Cerambycidae | Asia | Crating | Slate |
| Cerambycidae | Cerambycinae | Asia | Crating | Marble |
| Cerambycidae | *Monochamus* sp. | Europe | Crating | Rock |
| Cerambycidae | *Monochamus* sp. | Europe | No data | Tiles |
| Cerambycidae | *Tetropium castaneum* | Europe | Crating | Tiles |
| Cerambycidae | *Tetropium* sp. | Europe | Pallet | Tiles |
| Cerambycidae | *Xylotrechus rusticus* | Europe | Crating | Marble |
| Scolytidae | *Hylurgus ligniperda* | Europe | Crating | Tiles |
| Scolytidae | *Pityophthorus* sp. | Cen Amer | Crating | Prickly pear |
| Scolytidae | *Pityophthorus* sp. | Cen Amer | Pallet | No data |
| Scolytidae | Scolytidae | Cen Amer | Pallet | Leucaena fruit |
| Scolytidae | Scolytidae | Cen Amer | Pallet | Clothing |
| Scolytidae | Scolytidae | Cen Amer | Crating | Pepper |
| **Interceptions made during phase-in period of ISPM 15 (2005-2006)** | | | | |
| Cerambycidae | *Arhopalus* sp. | Europe | Crating | Tiles |
| Cerambycidae | Cerambycidae | Europe | Crating | Tiles |
| Cerambycidae | Cerambycidae | Europe | Dunnage | Cable |
| Scolytidae | Scolytidae | Cen Amer | Pallet | Broccoli |
| Scolytidae | Scolytidae | Cen Amer | Pallet | Epazote |
| **Interceptions made Post-ISPM 15 (2006-2009)** | | | | |
| Cerambycidae | *Arhopalus* sp. | Europe | Pallet | Tiles |
| Cerambycidae | Cerambycidae | Asia | Crating | Electric motor |
| Cerambycidae | Cerambycidae | Asia | Crating | Granite |
| Cerambycidae | Cerambycidae | Asia | Crating | Machinery |
| Cerambycidae | Cerambycidae | Europe | Crating | Tile |
| Cerambycidae | Cerambycidae | Europe | Pallet | Tiles |
| Cerambycidae | Cerambycidae | S Amer | Pallet | Tiles |
| Cerambycidae | Cerambycinae | Asia | Pallet | No data |
| Cerambycidae | Cerambycinae | Asia | Pallet | No data |
| Cerambycidae | *Monochamus* sp. | Asia | Pallet | Auto parts |
| Cerambycidae | *Monochamus* sp. | Asia | Pallet | Glass |
| Cerambycidae | *Monochamus* sp. | Asia | Pallet | Tiles |
| Cerambycidae | *Tetropium* *castaneum* | Europe | Crating | Cable |
| Cerambycidae | *Xylotrechus* *magnicollis* | Asia | Crating | Stone |
| Cossidae | Cossidae | Asia | Crating | Marble |
| Platypodidae | Platypodidae | S Amer | Pallet | Copper coil |
| Scolytidae | *Ips* sp. | Cen Amer | Pallet | Broccoli |
| Scolytidae | *Ips* *sexdentatus* | Europe | Pallet | Tiles |
| Scolytidae | *Pityophthorus* sp. | Cen Amer | Pallet | Tomatillo |
| Scolytidae | Scolytidae | Asia | Crating | Tiles |
| Scolytidae | Scolytidae | Asia | Crating | No data |
| Scolytidae | Scolytidae | Asia | Dunnage | Granite slab |
| Scolytidae | Scolytidae | Cen Amer | Pallet | Banana |
| Scolytidae | Scolytidae | Cen Amer | Pallet | Chinese cabbage |
| Scolytidae | Scolytidae | Cen Amer | Pallet | No data |
| Scolytidae | Scolytidae | Cen Amer | Pallet | Papaya |
| Scolytidae | Scolytidae | Cen Amer | Pallet | Pepper |
| Scolytidae | Scolytidae | Europe | Crating | Marble slab |
| Scolytidae | Scolytidae | S Amer | Crating | Electrolyte |
| Scolytidae | *Xyleborus* *eurygraphus* | Europe | Crating | Tiles |

^a^ Family names are presented as they appeared in the AQIM database. The authors recognize that the bark and ambrosia beetle family Scolytidae is now considered a subfamily of Curculionidae.

^b^ Cen Amer = Central America including Mexico, S Amer = South America.
